# Digital Impedance Emulator for Battery Measurement System Calibration

**DOI:** 10.3390/s21217377

**Published:** 2021-11-06

**Authors:** Francesco Santoni, Alessio De Angelis, Antonio Moschitta, Paolo Carbone

**Affiliations:** Engineering Department, University of Perugia, 06125 Perugia, Italy; alessio.deangelis@unipg.it (A.D.A.); antonio.moschitta@unipg.it (A.M.); paolo.carbone@unipg.it (P.C.)

**Keywords:** battery management, impedance spectroscopy, impedance emulator, digital filter, fir filter, instrument calibration

## Abstract

Meaningful information on the internal state of a battery can be derived by measuring its impedance. Accordingly, battery management systems based on electrochemical impedance spectroscopy are now recognized as a feasible solutions for online battery control and diagnostic. Since the impedance of a battery is always changing along with its state of charge and aging effects, it is important to have a stable impedance reference in order to calibrate and test a battery management system. In this work we propose a programmable impedance emulator that in principle could be used for the calibration of any battery management system based on electrochemical impedance spectroscopy. A digital finite-impulse-response filter is implemented, whose frequency response is programmed so as to reproduce exactly the impedance of a real battery in the frequency domain. The whole design process of the filter is presented in detail. An analytical expression for the impedance of real battery in the frequency domain is derived from an equivalent circuit model. The model is validated both through numerical simulations and experimental tests. In particular, the filter is implemented on a low-cost microcontroller unit, and the emulated impedance is measured by means of a custom-made electrochemical impedance spectroscopy measuring system, and verified by using standard commercial bench instruments. Results on this prototype show the feasibility of using the proposed emulator as a fully controllable and low-cost reference for calibrating battery impedance measurement systems.

## 1. Introduction

Measuring the impedance of a battery is being increasingly recognized as a fundamental step for its online diagnostic [1,2], i.e., when it is connected and operating in any battery-powered electric/electronic system. It is well known that Electrochemical Impedance Spectroscopy (EIS) data can provide meaningful information on the internal state of a battery [3,4]. Different portions of the impedance curve in the frequency domain are indeed related to different internal components and processes that can be modeled by means of equivalent circuits, and that are correlated with the battery State-of-Charge (SOC) and the State-of-Health. Yet as much as this topic has been widely investigated, most of the published experimental results are obtained by means of standard laboratory bench equipment that is not suited for online applications [5]. Online battery control and diagnostic systems, standardly referred to as Battery Management Systems (BMSs), are a key component in many applications, the automotive sector in particular being presently the subject of extensive research [6,7]. The integration of online EIS measurements into BMSs is an important development that is currently being investigated by several authors, with some promising solutions that have already been published [8,9,10,11,12].

Calibration and test under several working conditions are unavoidable stages in the developing process of a BMS [13,14]. The use of real batteries at this stage is not feasible, since the internal state of a battery and its response to external signals and solicitations change among different batteries (even of the same brand and model). Moreover, the characteristics of a single battery are not stable enough when repeated measurements are performed. A fully controllable reference is thus needed. Commercial bench instruments, such as the Keithley 2281S Series or ITECH IT6400 Series, are DC power supplies allowing for both current sourcing and sinking, which can simulate the voltage of a battery according to a predefined discharge curve [15]. These instruments are well suited to test charge and discharge cycles, but they do not emulate the impedance of a real battery and cannot be used to simulate the time transients expected in operating conditions.

A BMS testing approach frequently discussed in the literature is based on the so-called Hardware-In-the Loop paradigm (HIL) [16,17,18]. In general, a HIL simulator is a hardware that emulates all the input and outputs of the actual system under consideration. In this case, the HIL is designed to emulate a battery or a battery pack, its voltage, current, frequency response, state-of-charge, aging and failures. The BMS under test is interfaced to the HIL, and it can then be operated as if it were connected to an actual battery pack. The HIL is a piece of hardware fast enough to calculate and reproduce in real time the outputs of a real battery according with all the external inputs and solicitations. In general, a HIL simulator is a complex modular system that has to be designed and assembled for specific operations and requirements. Widely used commercial equipment is provided by dSPACE GmbH. In particular, the dSPACE EV1077 emulation board can emulate four cells and in principle can be programmed according to any mathematical model, such as equivalent circuit models including temperature and aging effects. Less complex and even low-cost solutions have also been presented in the literature [19,20,21].

In [22], a programmable setup implementing impedance emulation is proposed for the calibration of LCR meters. A power supply is used to sink the current supplied by the LCR meter, while a voltage generator and a current generator are used to emulate the voltage drop *V* generated by a current *I* across an arbitrary impedance *Z*. Both *V* and *I* are then measured by the LCR-meter in order to estimate $Z=\frac{V}{I}$. In this work, we propose an even simpler method to emulate the impedance of a battery that can be used as a reference to calibrate an EIS based BMS or other EIS equipment. The basic idea is to program a Finite Impulse Response filter (FIR), so that its frequency response would match exactly the impedance of the battery that has to be emulated. A general overview of the method is presented in Section 2. An experimental realization will be presented in Section 4.

The main difference of the proposed emulator with respect to the other reviewed solutions is that it does not source or sink any current. As regards the advantages, the emulator can be simply connected in place of the battery to the EIS equipment. It is programmable virtually with any impedance curve, so as to provide a reference for any state of a real battery. Finally, it is a low-cost solution requiring minimal equipment and components, that can be easily replicated to emulate multiple batteries.

## 2. General Overview of the Impedance Emulation Method

**Figure 1:**
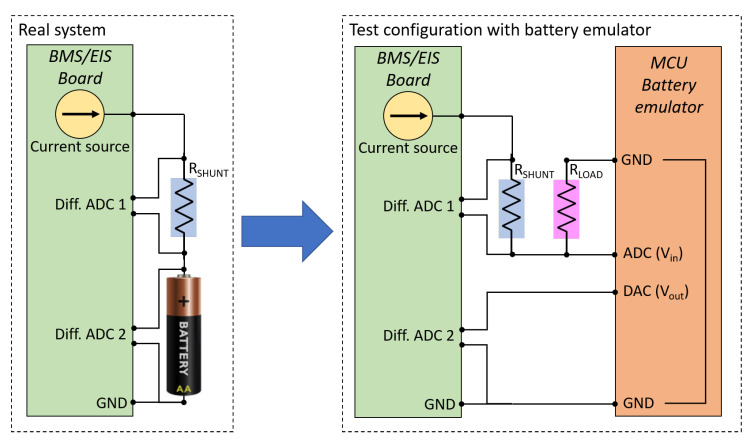
(**Left**): The scheme of the actual EIS measurement system connected to a battery. (**Right**): the same EIS instrument, but connected to the battery emulator implemented by means of a microcontroller unit provided with a unipolar ADC and a DAC.

We refer to the BMS/EIS equipment presented in [11], since it is a very simple system possibly representing the basic scheme of a class of BMSs to be developed. The reference EIS equipment is illustrated in Figure 1. It essentially consists of a controllable current source and two differential Analog-to-Digital Converters (ADCs). A controlled current *I* is injected into the battery through a shunt resistor $R_{shunt}$ of known value. The injected current is estimated by measuring with ADC1 the voltage difference $V_{shunt}$ across the shunt. ADC2 measures the voltage difference $V_{out}$ across the battery. Thus, the complex impedance can be obtained as

(1) $$Z\left(\omega \right)=\frac{V_{out}\left(\omega \right)}{I\left(\omega \right)}=R_{shunt}\frac{V_{out}\left(\omega \right)}{V_{shunt}\left(\omega \right)},$$

where ω is the angular frequency in rad/s.

**Figure 2:**
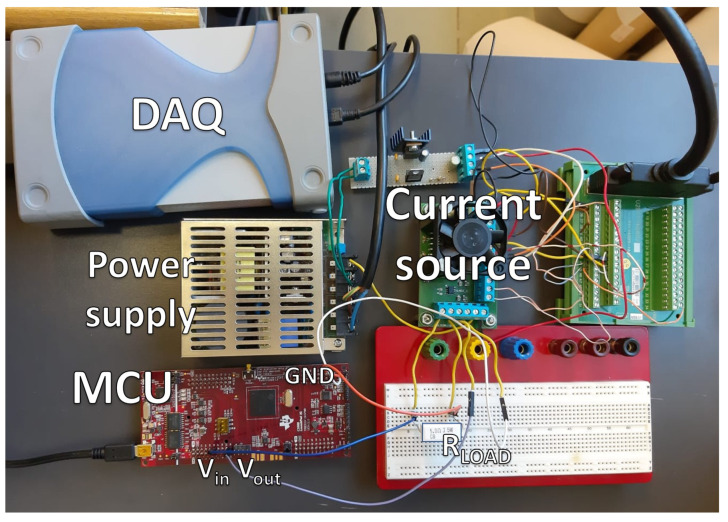
A picture of the prototype EIS measurement instrument and the impedance emulator. The EIS instrument consists of a custom-made current source with its power supply, and a Data Acquisition board (DAQ) that provides two differential ADCs (cf. Figure 1) and is also used to control the current source. The impedance emulator is implemented on a Texas Instruments MCU development board. A simple breadboard is used to connect the shunt resistor and to interface the BMS to the impedance emulator.

The impedance emulator, sketched on the right of Figure 1, is designed to be connected in place of the real battery, with the only difference that the current *I* is not injected into the emulator, but flows directly towards the ground through a load resistor $R_{load}$. By means of an ADC, the emulator acquires the voltage $V_{in}$ across the load, and outputs a voltage $V_{out}$ through a Digital-to-Analog Converter (DAC). A Microcontroller Unit (MCU) is programmed to generate $V_{out}$ according with a predefined impedance model. A picture of the built prototype is shown in Figure 2.

The MCU acts as a digital FIR filter, acquiring $V_{in}$ at a sample rate $F_{s}=\frac{1}{T_{s}}$, obtaining as input and output the discrete time sequences $x\left[n\right]=V_{in}\left(nT_{s}\right)$ and $y\left[n\right]=V_{out}\left(nT_{s}\right)$, which are related as:

(2) $$y\left[n\right]=\sum_{k=0}^{N-1}h\left[k\right]x\left[n-k\right],$$

where $h\left[n\right]$ is the impulse response of the system in the time domain, and *N* is the total number of samples. It is well known from the theory of digital filters [23] that $h\left[n\right]$ is related to the frequency response of the system $Z\left(\omega \right)$ by the Discrete Fourier Transform (DFT) as:

(3) $$Z\left(\omega_{k}\right)=\sum_{n=0}^{N-1}h\left[n\right]e^{-i\frac{2\pi kn}{N}},$$

where $\omega_{k}=\frac{2\pi F_{s}}{N}k$. Thus, the MCU is programmed so as to keep the ratio of $V_{out}\left(\omega \right)$ over $V_{in}\left(\omega \right)$ always equal (numerically) to the battery impedance $Z\left(\omega \right)$ that one wants to emulate, independently of the value selected for $R_{load}$. When the emulated impedance is measured by means of the EIS equipment, the following relations hold:

(4) $$Z\left(\omega \right)=\frac{V_{out}\left(\omega \right)}{V_{in}\left(\omega \right)}=\frac{V_{out}\left(\omega \right)}{R_{load}I\left(\omega \right)}=\frac{R_{shunt}}{R_{load}}\frac{V_{out}\left(\omega \right)}{V_{shunt}\left(\omega \right)}.$$

Thus, once programmed, the same emulator could be in principle adapted to different EIS instruments by just selecting a suitable $R_{load}$.

A reference impedance model $Z\left(\omega \right)$ has to be chosen. It can be obtained from the measured impedance of a real battery as it will be illustrated below. Then, the coefficients $h\left[n\right]$ are obtained by inverting the DFT and stored in the non-volatile memory of the MCU. Each time a sample of $V_{in}$ is acquired by the ADC, the convolution sum (2) is calculated, and the resulting $V_{out}$ is written on the DAC register. The length of the sequence *N* is upper bounded by the memory capacity, and also by the clock frequency of the MCU, since the convolution sum has to be computed in a time shorter than the sampling period $T_{s}$; *N* is also lower bounded in accordance with the frequency resolution $\Delta f=\frac{F_{s}}{N}$ required by any particular application. The full design process of the FIR filter is illustrated step-by-step in Section 3, while the experimental implementation of a test prototype is presented in Section 4.

## 3. Design of the Fir Filter

The impedance emulating FIR filter has been designed by going through the following steps. A paragraph will be devoted to the details of each step.

1. *Modeling the impedance of a battery*. Since the aim of the filter is to emulate a real battery, it has been designed to reproduce an experimental impedance. An analytical model facilitates the design process, and allows for more control, thus, the measured impedance has been fitted to an equivalent circuit model.
2. *Choosing the number of samples and the sampling rate*. This has to be done according to the frequency range of interest for the impedance, and with the memory capacity and the clock frequency of the MCU.
3. *Definition of the impulse response*. It has been derived from the impedance curve in the frequency domain by an Inverse DFT.
4. *A numerical simulation of the filter response*. This has been performed on MATLAB in order to verify that the model is correct.

### 3.1. Step 1: Modeling the Impedance of the Battery

As reference, we used the battery model ICR18650-26J by Samsung, that had been already used to test our custom EIS equipment in [11]. The chosen excitation current signal was a multisine whose frequency components were logarithmically spaced: [0.1, 0.2, 0.4, 1, 2, 4, 10, 20, 40, 100, 200, 400] Hz. We measured the impedance $Z\left(\omega \right)$ for SOC 100% and 20%.

**Figure 3:**
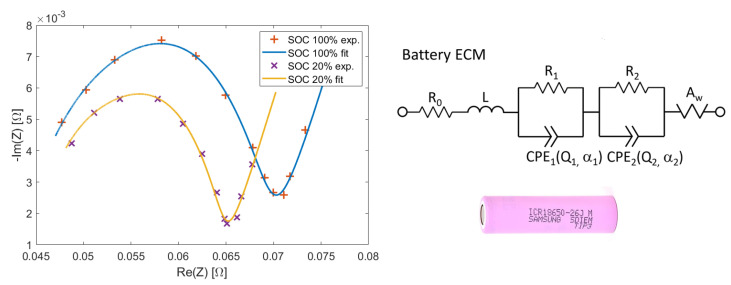
Measured impedance curve of the Samsung battery at SOC 100% and 20%. The equivalent circuit model shown on the right has been fitted to experimental data. A picture of the actual battery is also shown.

The experimental results are shown in Figure 3. The choice of the two SOCs is motivated by the fact that they are associated with two impedance curves that are significantly different, practically being at the extremes of the range of variation of the battery impedance. Hence, we wanted to check the performance of the system at both the extremes (see Section 5.1). The battery has been modeled as the equivalent circuit shown in the same figure. It is a model commonly used in the literature, which we had previously verified to fit well the experimental impedance curves of the Samsung battery [24]; it implements some fractional order components, i.e., two Constant Phase Elements (CPEs) and a Warburg element. The complex impedance of the equivalent circuit is:

(5) $$Z\left(s\right)=R_{0}+sL+\frac{R_{1}}{1+R_{1}Q_{1}s^{\alpha_{1}}}+\frac{R_{2}}{1+R_{2}Q_{2}s^{\alpha_{1}}}+\frac{\sqrt{2}A_{w}}{s^{0.5}},$$

where s=iω. The model has thus nine parameters $\theta =\left[R_{0},L,R_{1},Q_{1},\alpha_{1},R_{2},Q_{2},\alpha_{2},A_{w}\right]$ that have to be fitted to experimental data. The solid curves plotted in Figure 3 have been calculated by using Formula (5), after θ had been estimated through a non-linear least-squares fitting algorithm. The chosen frequency interval allows detection of the two main features of the impedance curve: the semicircle at higher frequencies and the straight line at 45${}^{\circ }$ at lower frequencies.

### 3.2. Step 2: Choosing the Number of Samples and the Sampling Rate

The number of samples *N* and the sampling rate $F_{s}$ cannot be selected independently, since they both define the frequency resolution as $\Delta f=\frac{F_{s}}{N}$. The frequency range of interest is 0.1–400 Hz, hence, in order to meet the Nyquist condition, the sampling rate should be $F_{s}>800$ Sa/s. We chose $F_{s}=1000$ Sa/s. Given the memory capacity of the MCU used to implement the prototype (see the experimental Section 4), we set *N* = 30,000 Sa. Thus, the frequency resolution of our impedance emulator is Δf=33 mHz, which is enough to even discriminate between the two lower frequency components (100 and 200 mHz). On the chosen MCU, the computation of (2) with *N* = 30,000 requires 827 μs, which is compatible with the chosen sampling period $T_{s}=1$ ms. In case of a different frequency range of interest or different MCU for other applications, the parameters should be reconfigured accordingly to the following analogous criteria. The parameters of the prototype configuration are summarized in Table 1 in the experimental Section 4.

### 3.3. Step 3: Definition of the Impulse Response

By construction, the frequency response of the FIR filter (i.e., numerically, the impedance that we want to emulate) is the DFT of the impulse response $h\left[n\right]$. Thus, by means of (5), the impedance $Z\left(f_{k}\right)$ can be calculated for the frequency values ${\left\{f\right\}}_{k}=\frac{F_{s}}{N_{s}}\left[0,1,\ldots ,k,\ldots ,\frac{N}{2}\right]$ (i.e., *N* equally spaced frequency values in the range 0–$\frac{F_{s}}{2}$), then the coefficients $h\left[n\right]$ are calculated by performing the inverse DFT of $Z\left(f_{k}\right)$ and stored in the non-volatile memory of the MCU.

A problem arises with the impedance of the Warburg element $Z_{w}\left(s\right)=\frac{\sqrt{2}A_{w}}{s^{0.5}}$, since it is not defined for $f_{0}=0$. Thus, the inverse DFT

(6) $$h\left[n\right]=\frac{1}{N}\sum_{k=0}^{N-1}Z\left(f_{k}\right)e^{i\frac{2\pi kn}{N}n}$$

cannot be computed. The solution is to use the low-frequency approximation of $s^{-0.5}$ given in [25]:

(7) $$Z_{i}=\sqrt{2}A_{w}\frac{s^{4}+36s^{3}+126s^{2}+84s+9}{9s^{4}+84s^{3}+126s^{2}+36s+1},$$

i.e., the fractional order response is approximated with an integer order one. Let us define:

(8) $${\tilde{Z}}_{w}\left(f\right)=\left\{\begin{array}{cc} Z_{i}\left(f\right) & f<1\mathrm{Hz}\\ Z_{w}\left(f\right) & f\geq 1\mathrm{Hz} \end{array}\right.$$

**Figure 4:**
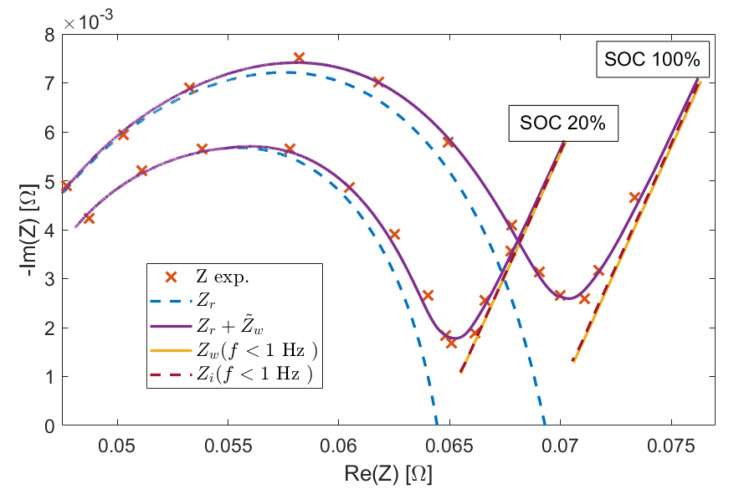
FIR frequency response obtained by using the approximation (7) compared with the experimental data. The components $Z_{r}$, $Z_{w}$ and $Z_{i}$ are also reported as to show the goodness of the approximation $Z_{w}\approx Z_{i}$ for f<1 Hz.

Hence, by defining $Z_{r}=Z-Z_{w}$, the impedance can be approximated as $Z\approx Z_{r}+{\tilde{Z}}_{w}$. A very good approximation is thus obtained, as shown in Figure 4.

### 3.4. Step 4: A Numerical Simulation of the Filter Response

The emulator model has been simulated with MATLAB in order to verify that numerical and discretization errors, as well as the noise will not significantly affect the results. Since on the MCU all the computations will be performed using 32-bit single-precision representation of numbers, in MATLAB the 32-bit precision was also explicitly selected, the 64-bit precision being the default.

Both the signal acquisition with the EIS equipment, and the acquisition and processing of the emulator have to be simulated. The simulation can be divided into three stages:

1. A simulated signal $V_{in}$ is acquired by the EIS equipment at a sampling rate $F_{aq}$, and by the emulator at sampling rate $F_{s}$. ADC signal quantization is simulated;
2. The signal $V_{out}$ is computed by summing (2), and the DAC output is synthesized as a Zero-Order-Hold signal (ZOH);
3. The acquisition of $V_{out}$ by the EIS equipment at sampling rate $F_{aq}$ is simulated. The impedance is estimated as the ratio of the DFTs of $V_{out}$ and $V_{in}$.

#### 3.4.1. Simulation Stage 1

**Figure 5:**
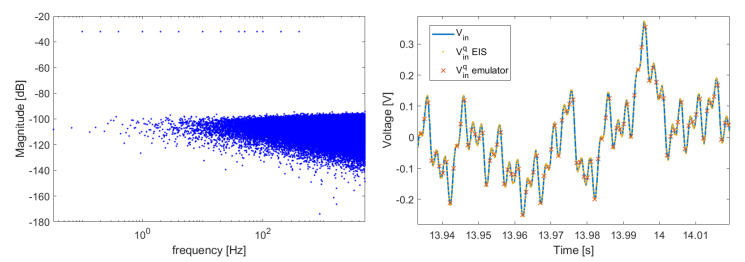
(**Left**): The power spectrum of the generated $V_{in}$. (**Right**): A portion of $V_{in}$ in the time domain. The sampled and quantized sequences are renormalized for ADC ranges and levels, in order to be shown together with the original signal.

Sampling rates and ADC ranges are reported in Table 1 and Table 2 on the experimental Section 4. $F_{s}=1$ kSa/s, and initially $F_{aq}=10$ kSa/s. The acquisition window is 30 s (number of samples $N_{aq}$ = 300,000). A multisine signal $V_{in}$ was generated with frequency components [0.1, 0.2, 0.4, 1, 2, 4, 10, 20, 40, 50, 80, 100, 200, 400] Hz. The amplitude of each component is 50 mV. Signal quantization was simulated as:

(9) $$V_{in}^{q}=\left\lfloor \left(V_{in}+\epsilon \right)\frac{\mathrm{ADC}\;\mathrm{range}}{\mathrm{ADC}\;\mathrm{levels}}\right\rfloor ,$$

where ⌊⌋ indicates the *floor* function, while ϵ is a zero-mean Gaussian noise with variance $\sigma^{2}$. Simulations were performed by setting σ=0 and 3 mV. The second value for the noise was used since it is comparable to the random noise observed on experimental measurements. The simulated input signal is shown in Figure 5 for σ=3 mV.

#### 3.4.2. Simulation Stage 2

**Figure 6:**
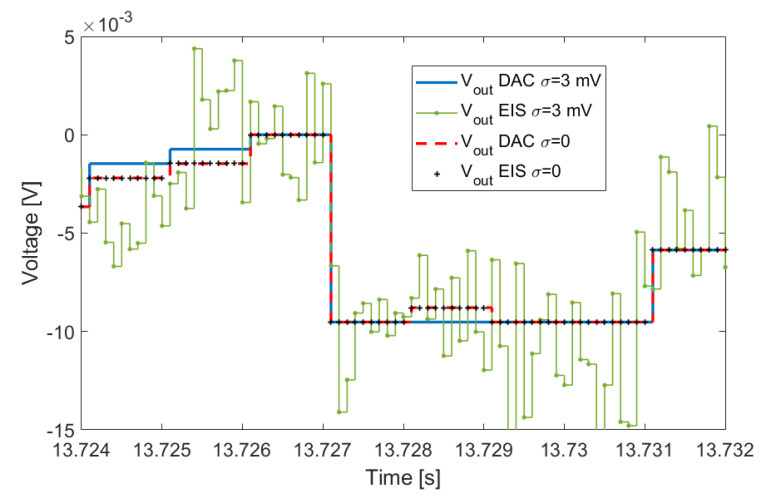
$V_{out}$ resulting from (2) simulated as a zero-order-hold signal generated by the DAC and sampled with the ADC2 of the EIS instrument. See Section 3.4.2 for more details.

The output $V_{out}$ resulting from (2) is shown in Figure 6. The solid blue line is the ZOH signal generated by the DAC with period $T_{s}$, and σ=3 mV. The green line with dot markers is the simulation result of the DAC signal as acquired by the EIS measurement system with sampling period $T_{aq}=10T_{s}$. Additionally, the ADC quantization for $V_{out}$ was simulated as it was done for $V_{in}.$ The analogous results for σ=0 are also reported in the plot. It can be noted that $V_{out}$ as synthesized by the DAC is very stable when going from σ=0 to σ=3 mV; this is explained by the fact that $V_{out}$ is approximately an order of magnitude lesser than $V_{in}$, hence the noise acquired with $V_{in}$ is reduced accordingly, and it almost disappears given the finite resolution of the DAC. Then, the noise is added again as the DAC signal is re-acquired by the EIS system.

#### 3.4.3. Simulation Stage 3

The frequency spectra of both input and output signals are obtained by applying a Fast Fourier Transform (FFT) to the sampled sequences of measured voltages: $V_{in}\left(f_{k}\right)=FFT\left(V_{in}^{q}\left[n\right]\right)$ and $V_{out}\left(f_{k}\right)=FFT\left(V_{out}^{q}\left[n\right]\right)$, where ${\left\{f\right\}}_{k}=\frac{F_{aq}}{N_{aq}}\left[0,1,\ldots ,k,\ldots ,\frac{N_{aq}}{2}\right]$. The spectrum of $V_{out}$ has to be corrected for the distortion introduced by the ZOH as

(10) $$V_{out}\left(f_{k}\right)\to V_{out}\left(f_{k}\right)\exp\left[i2\pi f_{k}\left(T_{s}-T_{aq}\right)\right]\frac{\mathrm{sinc}\left(f_{k}T_{aq}\right)}{\mathrm{sinc}\left(f_{k}T_{s}\right)}.$$

This correction is explained and formally derived in Appendix A. The ratio between the corrected $V_{out}\left(f_{k}\right)$ (10) and $V_{in}\left(f_{k}\right)$ yields the FIR frequency response.

#### 3.4.4. Simulation Results and Discussion

**Figure 7:**
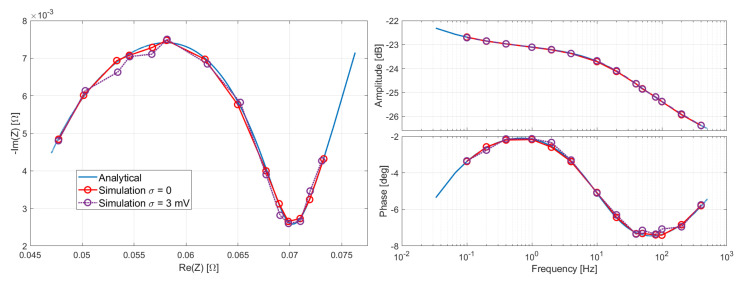
(**Left**): Nyquist plot of the simulated FIR frequency response compared with the analytical response. (**Right**): Bode plots of amplitude and phase of the FIR frequency response.

In Figure 7, the simulated response of the FIR filter is reported and compared with the analytical response function (5). For σ=0 the agreement is very good for both amplitude and phase. For σ=3 mV, the agreement is still very good for the amplitude, but some errors are introduced in the phase, in particular at higher frequencies f>20 Hz. Regardless, in the worst case, the relative error affecting $ℑm\left(Z\right)$ is 9%, while the mean relative error is 3%. The relative error on $ℜe\left(Z\right)$ is negligible, its mean value being 0.2%, and 0.4% in the worst case.

**Figure 8:**
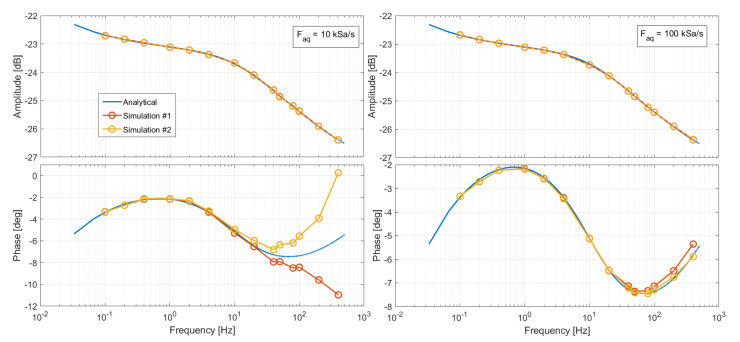
Effects of the non-synchronization of the EIS board with the emulator board. A random delay or anticipation has been inserted between $V_{in}$ and $V_{out}$ in the simulation. (**Left**): Simulation results for amplitude and phase of the FIR frequency response when the sampling period of the EIS board is set to $T_{aq}=100$ μs. (**Right**): Simulation results when $T_{aq}=10$ μs.

The simulation was performed assuming that signal acquisitions on the EIS measurement system and on the emulator are perfectly synchronous. Although it would be possible, in principle, to implement the synchronization on the actual system, the emulator is intended to be a portable instrument applicable to different equipment, also when the synchronization is not possible. When the two systems are not synchronized, the sampling instant of the emulator can fluctuate with a flat distribution within the $T_{aq}$ sampling period of the EIS equipment. This results in a random delay or anticipation between $V_{in}$ and $V_{out}$ affecting the phase of the impedance. Several simulations have been performed by including between $V_{in}$ and $V_{out}$ a random delay Δt in the interval $\left(-\frac{T_{aq}}{2},\frac{T_{aq}}{2}\right)$. The Bode plots of the frequency response for a couple of simulations are shown in Figure 8. The response amplitude is not affected at all, but for $F_{aq}=10$ kSa/s, the phase measured at higher frequencies is not repeatable because of the random delay fluctuations. The only solution to this problem, if synchronization is not feasible, is to use a higher sampling frequency, such as $F_{aq}=100$ kSa/s, as shown on the right of Figure 8.

## 4. Experimental Implementation

### 4.1. Implementation of the Impedance Emulator on an MCU

The impedance emulation method described above could be in principle implemented on any MCU equipped with an ADC and a DAC, provided that its specifications such as clock frequency, ADC sampling rate, resolution, and so on, meet the requirements of the particular application it is intended to be used for. In order to test the devised method, we used the low-cost development board LAUNCHXL-F28379D by Texas Instruments, mounting the 32-bit, 200 MHz dual-core microcontroller TMS320F28379D. All the relevant specification and settings of this implementation are summarized in Table 1. Important configuration details have already been discussed on Section 3.2. Here we add that the duration of the ADC sample and hold window had to be set at least to 125 μs as reported, otherwise, for shorter times, the acquired signal was to noisy, resulting in unacceptable measurement errors.

**Figure 9:**
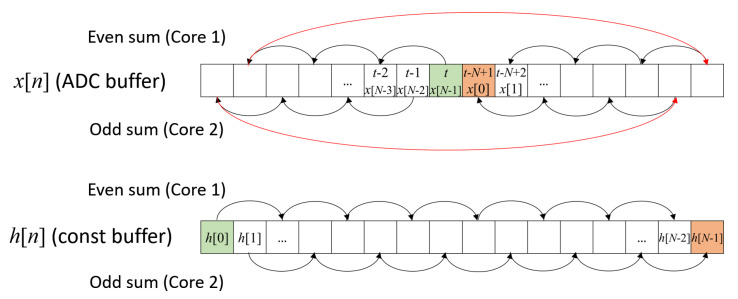
The sequence of the iterations performed to compute the convolution (2), split into two separate sums over the even and odd terms, respectively. The first term of the summation is indicated in green, that is, the memory location storing the last acquired sample, i.e., the sample at current time *t*. The last term is indicated in red.

The computation of the convolution sum (2) was performed as follows. The ADC buffer consists of *N* 16-bit unsigned integer memory locations storing the sequence $x\left[n\right]$, while the impulse response $h\left[n\right]$ is stored in a constant buffer consisting of *N* 32-bit floating point memory locations. In order to store the continuously updating $x\left[n\right]$, circular buffering was used, i.e., a pointer to an address of the ADC buffer is incremented by one at each acquisition, and when the end of the buffer is reached, the pointer returns to the beginning of the buffer; hence, after *N* acquisitions, older values start to be overwritten, since they are not needed anymore. In order to exploit the dual-core parallelism of the MCU, the convolution was split into two separate sums, one over the even terms, computed by core 1, and one over the odd terms, computed by core 2. The sequence of the iterations performed for both sums is illustrated in Figure 9, starting from the memory location labeled as *t* storing the last acquired sample.

### 4.2. The Acquisition System

The DAQ of our custom EIS equipment is the 16-bit U2351A data acquisition board from Keysight. The setting we used to test the impedance emulator by acquiring $V_{in}$ and $V_{out}$ are summarized in Table 2. The application of the FFT and the computation of the impedance, as illustrated on Section 3.4.3, are performed in post-processing by using MATLAB. No windowing has been applied, since the sampling rates and the number of samples were chosen as to always acquire an integer number of periods of each sinusoidal component [11]. The DAQ board also mounts a DAC that we used to pilot the voltage-controlled current pump in order to generate the multisine excitation signal. The amplitude of each current component was 50 mA. The shunt and load resistor values were $R_{shunt}=200$ mΩ, and $R_{load}=5$Ω.

In order to check the reproducibility of the results, we also tested the emulator by using a bench waveform generator to generate $V_{in}$, and a bench oscilloscope to acquire $V_{in}$ and $V_{out}$. Resistors $R_{shunt}$ and $R_{load}$ were not used in this phase. The settings of the oscilloscope are reported in Table 2. The clocks of the two instruments were synchronized by wiring the oscilloscope external-clock input connector to the clock output connector of the waveform generator.

After some preliminary measurements, and by following the analysis presented in Section 3.4.4, we decided to measure the emulated impedance in two separate stages, one for the lower and one for the higher frequencies. Indeed, at lower frequencies, a longer acquisition time is needed in order to acquire several periods of the slower sinusoidal components, hence, the sampling rate $F_{aq}=10$ kSa/s was used in order to limit the total number of samples. At frequencies greater than 20 Hz, we instead used the sampling rate $F_{aq}=100$ kSa/s, in order to avoid phase fluctuations of the kind shown in Figure 8. The results were then combined into a unique impedance curve over the whole frequency interval 0.1–400 Hz.

As a last point, in simulations, $V_{out}$ was treated as it were generated instantly as $V_{in}$ was acquired by the ADC of the emulator. Of course, this is not the case in the real system, that has instead a latency due to the time required to compute (2), introducing a delay $T_{c}=827$ μs between $V_{in}$ and $V_{out}$. Since the Laplace transform of a delayed function is simply:

(11) $$L\left[f\left(t-T_{c}\right)\right]\left(s\right)=\exp\left(-sT_{c}\right)L\left[f\left(t\right)\right]\left(s\right),$$

the effect of the MCU latency on the impedance $Z\left(f_{k}\right)$ is corrected by just multiplying $V_{out}\left(f_{k}\right)$ by the phase factor $\exp\left(i2\pi f_{k}T_{c}\right)$.

## 5. Results and Discussion

### 5.1. Results Obtained on the Custom EIS Measurement System

**Figure 10:**
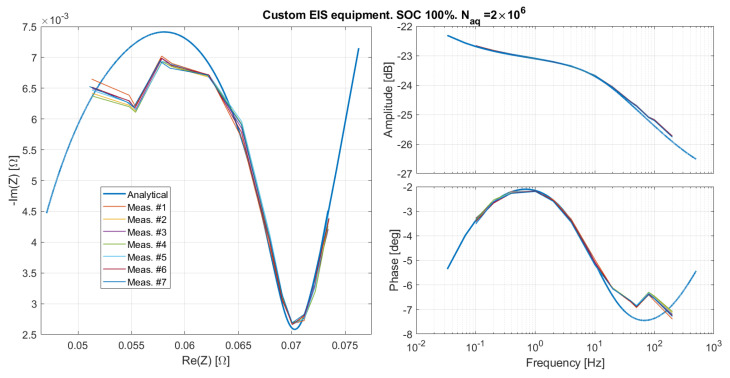
The emulated impedance as measured by the custom EIS system for seven repeated measurements.

The emulated impedance for the SOC 100%, as measured by means of the custom EIS equipment, is reported in Figure 10 for seven repeated measurements. It can immediately be noticed that, although the fluctuations due to random noise are small, there are, however, considerable systematic phase distortions at higher frequencies. Points measured at frequencies greater then 100 Hz looks like complete outliers, while at smaller frequencies, it seems that a linear phase distortion $\exp\left(-i2\pi f_{k}T_{d}\right)$ is present. This can be verified by applying the following calibration procedure:

1. *Phase calibration*. Let us consider the column vector of the measured phases $\varphi_{m}={\left[\varphi \left(f_{0}\right),\varphi \left(f_{1}\right),\ldots \right]}_{m}^{T}$, where the index *m* is used to indicate each one of the repeated measurements, and the vector defined by arranging all the $\varphi_{m}$ in a single column, $\varphi ={\left[\varphi_{1},\varphi_{2},\ldots \right]}^{T}$. Let us also consider the analogous vector $\varphi_{0}$ of the expected phases computed analitically. Finally, the column vector f of all the frequencies ${\left\{f\right\}}_{k}$ repeated many times on a column as the number of repeated measurements (i.e., f, φ and $\varphi_{0}$ have the same number of elements). The difference between the measured and the expected phase can then be written as the following linear system in the single unknown $T_{d}$:
  (12) $$2\pi fT_{d}=\varphi_{0}-\varphi .$$
  The least-squares solution is $T_{d}=-29.7$ μs. Only the frequencies up to 100 Hz were included in the equation system.
2. *Amplitude calibration*. Since the ADCs of the acquisition system and of the emulator are different, and can produce different results on equal signals, in general, the amplitude has to be calibrated. As above, let us consider the column vector of the measured amplitudes $A_{m}={\left[A\left(f_{0}\right),A\left(f_{1}\right),\ldots \right]}_{m}^{T}$, then again the single column arrangement $A={\left[A_{1},A_{2},\ldots \right]}^{T}$, and finally the vector $A_{0}$ of the expected amplitudes computed analytically. The amplitude correction is given by a calibration constant $k_{A}$ determined by the following linear system:
  (13) $$Ak_{A}=A_{0}.$$
  The least-squares solution is $k_{A}=0.9938$, very close to 1. Indeed, in this case, as it can be seen in Figure 10, the amplitudes were already well matching the expected curve even without calibration.

**Figure 11:**
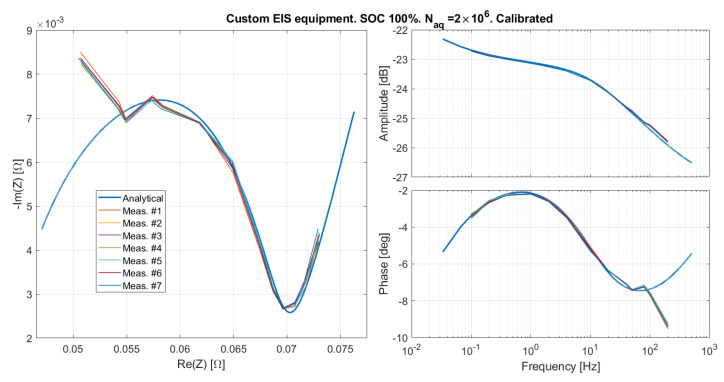
The emulated impedance as measured by the custom EIS measurement system after the calibration illustrated in Section 5.1.

**Figure 12:**
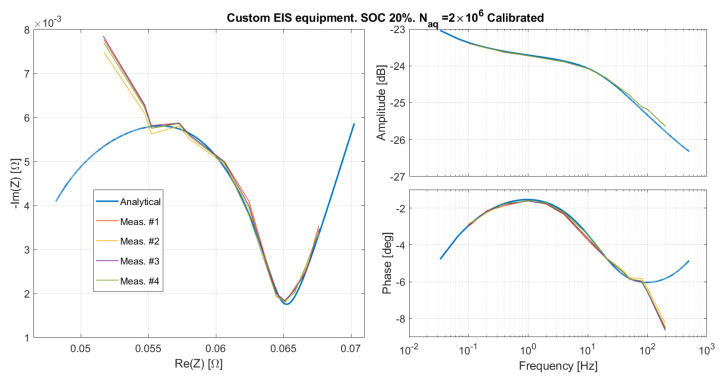
The emulated impedance as measured by the custom EIS measurement system after the calibration illustrated in Section 5.1.

By applying the calibration procedure, the corrected results shown in Figure 11 were obtained. The good agreement of the measured and expected curves up to 100 Hz proves that the hypothesis of a linear phase distortion was right. Nevertheless, the non-linear systematic phase distortion above 100 Hz remains. By keeping the values of $T_{d}$ and $k_{A}$ just computed, and measuring the emulated impedance for the SOC 20%, again a good matching is obtained, as shown in Figure 12. This suggests that the calibration is linked to the EIS system, and does not depend on the response programmed in the filter. In any case, the origin of such a strong phase distortion is not clear. In order to verify that it is due to the EIS system, and not to the emulator, we performed a set of measurements with a bench oscilloscope.

#### Results Obtained on the Oscilloscope

**Figure 13:**
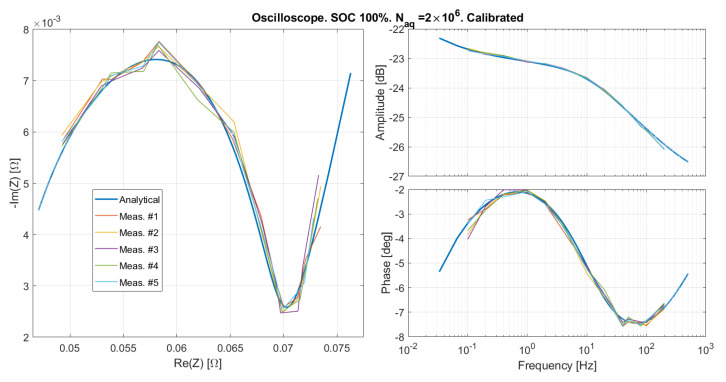
The emulated impedance as measured by means of a bench oscilloscope after the calibration illustrated in Section 5.1.

**Figure 14:**
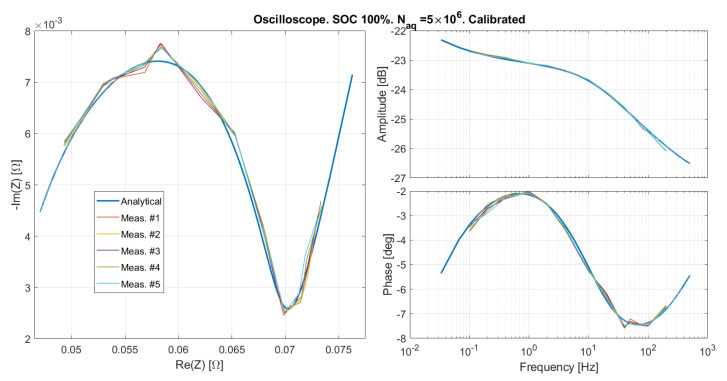
The emulated impedance as measured by means of a bench oscilloscope after the calibration illustrated in Section 5.1.

**Figure 15:**
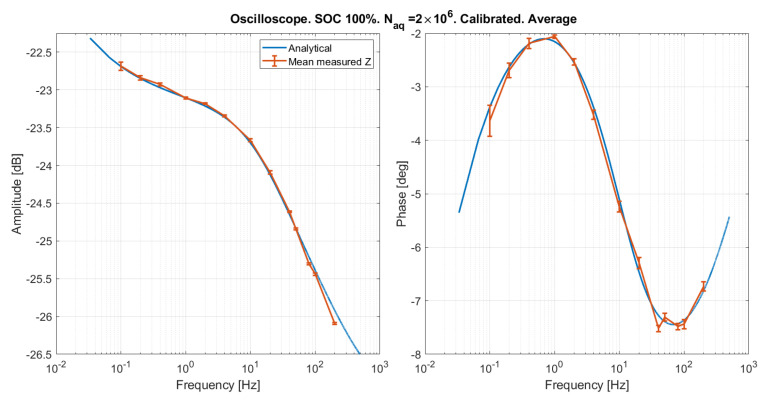
Mean and standard deviation over five repeated measurement of the emulated impedance.

**Figure 16:**
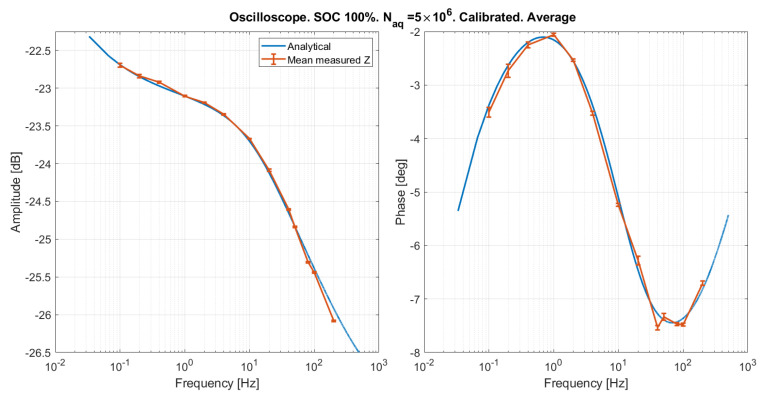
Mean and standard deviation over five repeated measurement of the emulated impedance.

The emulated SOC 100% was measured by means of the oscilloscope, and the same calibration procedure described in Section 5.1 was applied, obtaining $T_{d}=-3$ μs, and $k_{A}=1.02$. The results are reported for two different numbers of samples in Figure 13 and Figure 14, showing a good agreement between measured and expected curves. The slight phase distortion is within the time resolution set on the oscilloscope ($T_{aq}=10$ μs at higher frequencies), and might be due to the fact that there is no clock synchronization between the oscilloscope and the impedance emulator. The mean and standard deviation over five repeated measurements of the amplitude and phase of the emulated impedance are reported in Figure 15 and Figure 16. As expected, the error is lower for a greater number of samples. Systematic distortions are at any rate present also in the results obtained with the oscilloscope. However, the phase distortion above 100 Hz in this case is much smaller than that observed with the custom EIS system in Figure 13 and Figure 14. This indicates that the distortion at high frequency is due to the EIS system, and not to the emulator.

## 6. Conclusions

We presented a method to emulate the impedance of a battery by means of a digital filter. All the details of the design are described. A low-cost prototype of the impedance emulator has been implemented and tested. A good agreement between the programmed and measured impedance was obtained when the emulator was tested by means of a bench oscilloscope. A relevant non-linear phase distortion was instead observed when the emulator was tested on a custom EIS measurement system. Such distortion is clearly linked with the EIS equipment and will need further investigation. Additionally, other excitation signals (e.g., binary sequences [10]) still need to be investigated on the emulator.

Overall, however, results show the feasibility of using the proposed emulator as a fully controllable and low-cost reference for calibrating battery impedance measurement systems. An immediate continuation of this work will be the implementation of the emulator on a better performing MCU or on a single-board computer, in order to increase both the sampling rate $F_{s}$ and the number of samples $N_{s}$. This should allow emulation of the impedance at frequencies higher than 400 Hz, at the same time reducing the phase distortion.

## Figures and Tables

**Table 1:** Technical features of the impedance emulator prototype.

Feature	Value
ADC range	3 V, unipolar
ADC resolution	12 bit
ADC sampling rate $F_{s}$	1000 Sa/s
ADC sample & hold time	125 μs
DAC range	0–3 V
DAC resolution	12 bit
Number of samples *N*	30,000
CPU cores	2
CPU clock frequency	200 MHz

**Table 2:** Settings of the data acquisition system.

Feature	EIS Equipment/Keysight DAQ	Bench Oscilloscope
ADC1 range	5 V, bipolar	1.2 V, bipolar
ADC2 range	1.25 V, bipolar	0.12 V, bipolar
ADC1/2 resolution	16 bit	8 bit
ADC1/2 sampling rate $F_{aq}$	10 and 100 kSa/s	10 and 100 kSa/s
Number of samples $N_{aq}$	2 MSa	2 and 5 MSa

## Data Availability

The data presented in this study are available on request from the corresponding author. The data are not publicly available because they have not been ordered and stored in a clear and manageable form.

## Appendix A. Sampling the DAC Signal

First, we briefly review some basic notions on ZOH signals. Let $X\left(f\right)$ be the Fourier transform of any continuous time (c.t.) signal $x\left(t\right)$, and $X_{zoh}\left(f\right)$ the Fourier transform of its respective c.t. ZOH signal $x_{zoh}\left(t\right)$, sampled with period *T*. It is also assumed that the sampling rate F=1/T and the bandwidth of the c.t. signal satisfy the Nyquist criterion. Hence, the following relation holds [23]:

(A1) $$X_{zoh}\left(f\right)=X\left(f\right)\exp\left(-i\pi fT\right)\mathrm{sinc}\left(fT\right),$$

i.e., the ZOH signal has a delay of T/2 with respect to the original signal $x\left(t\right)$, and also its amplitude is distorted by the $\mathrm{sinc}\left(fT\right)$. In general, given the ZOH spectrum $X_{zoh}\left(f\right)$, the spectrum of the original signal $X\left(f\right)$ can be reconstructed by inverting (A1).

From now on, we always assume that the frequencies of all the spectral components, the sampling rates, and the duration of the acquisition window, are chosen in order to always acquire an integer number of cycles for each component, and that an integer number of samples is acquired at each cycle. Thus, the spectral components of the DFT with rectangular windowing, divided by the number of samples, are the same as the spectral components of the c.t. signals.

The DAC signal of Figure 6 is a c.t. ZOH with period $T_{s}$, generated from the sequence $V_{out}\left(nT_{s}\right)$ as computed by the FIR filter. Let us indicate as $V_{out}\left(nT_{aq}\right)$ the sequence obtained by sampling the DAC signal with sampling rate $F_{aq}=1/T_{aq}=$ 10 kHz (the EIS samples of Figure 6). To calculate the impedance, we need to calculate $\mathrm{DFT}\left[V_{out}\left(nT_{s}\right)\right]\left(f\right)$, where the sequence $V_{out}\left(nT_{s}\right)$ could be simply obtained by decimating $V_{out}\left(nT_{aq}\right)$. However, we want to keep all the samples, since, in general, this has an averaging effect on the noise, thus producing better results. We then derive a different method. One might think that (A1) could be inverted as

(A2) $$\frac{1}{N_{s}}\mathrm{DFT}\left[V_{out}\left(nT_{s}\right)\right]\left(f\right)=\frac{1}{N_{aq}}\mathrm{DFT}\left[V_{out}\left(nT_{aq}\right)\right]\left(f\right)\frac{\exp\left(i\pi fT_{s}\right)}{\mathrm{sinc}\left(fT_{s}\right)},$$

where $N_{aq}$ is the number of acquired samples, while $N_{s}=N_{aq}T_{aq}/T_{s}$ is the decimated number of samples. Although it is correct to identify $X\left(f\right)\equiv \mathrm{DFT}\left[V_{out}\left(nT_{s}\right)\right]\left(f\right)/N_{s}$, this inversion does not yield correct results. Indeed, $\mathrm{DFT}\left[V_{out}\left(nT_{aq}\right)\right]\left(f\right)/N_{aq}$ cannot be identified with the spectrum $X_{zoh}\left(f\right)$ of the c.t. ZOH signal of the DAC, since $X_{zoh}\left(f\right)$ is not band-limited. The two spectra might be considered the same when $T_{aq}\ll T_{s}$, and (A2) would thus be correct to a very good approximation. In any case, as we will see in the following, the problem has an exact solution, and it is not necessary to use an approximation, whatever the values of $T_{aq}$ and $T_{s}$ might be.

Intuitively, the solution can be derived as follows. The DAC output can be viewed as a c.t. ZOH signal with period $T_{aq}$ and constant for *N* periods, with $N=T_{s}/T_{aq}=N_{aq}/N_{s}$. Hence, in accordance with the general Formula (A1), its Fourier transform can be written as:

(A3) $$X_{zoh}\left(f\right)=\frac{1}{N_{aq}}\mathrm{DFT}\left[V_{out}\left(nT_{aq}\right)\right]\left(f\right)\exp\left(-i\pi fT_{aq}\right)\mathrm{sinc}\left(fT_{aq}\right).$$

Since, by construction, the c.t. ZOH signal with period $T_{aq}$ is the same as the c.t. ZOH signal with period $T_{s}$, their spectrum is the same, hence, we can invert (A1) as:

(A4) $$\begin{array}{c} \frac{1}{N_{s}}\mathrm{DFT}\left[V_{out}\left(nT_{s}\right)\right]\left(f\right)=X_{zoh}\left(f\right)\frac{\exp\left(i\pi fT_{s}\right)}{\mathrm{sinc}\left(fT_{s}\right)}=\\ =\frac{1}{N_{aq}}\mathrm{DFT}\left[V_{out}\left(nT_{aq}\right)\right]\left(f\right)\exp\left[i\pi f\left(T_{s}-T_{aq}\right)\right]\frac{\mathrm{sinc}\left(fT_{aq}\right)}{\mathrm{sinc}\left(fT_{s}\right)} \end{array}$$

that is the same as the correction (10) that we applied in Section 3.4.3. Let us now derive the same result more formally.

For the sake of brevity, we define $X_{s}\left(f\right)\equiv \mathrm{DFT}\left[V_{out}\left(nT_{s}\right)\right]\left(f\right)$, and $X_{aq}\left(f\right)\equiv \mathrm{DFT}\left[V_{out}\left(nT_{aq}\right)\right]\left(f\right)$. Let us also define the zero-padded sequence:

(A5) $$V_{out}^{zp}\left(mT_{aq}\right)=\left\{\begin{array}{cc} V_{out}\left(nT_{s}\right) & m=nN\\ 0 & \mathrm{otherwise} \end{array}\right.,$$

and let $X_{aq}^{zp}\left(f\right)$ be its DFT. It is a known result in the theory of signal processing that $X_{aq}^{zp}\left(f\right)=X_{s}\left(f\right)$[23]. The sequence $V_{out}\left(nT_{aq}\right)$ can be rewritten as a sum of translated zero-padded sequences:

(A6) $$V_{out}\left(mT_{aq}\right)=\sum_{k=0}^{N-1}V_{out}^{zp}\left[\left(m-k\right)T_{aq}\right],$$

and its DFT can thus be expressed as:

(A7) $$X_{aq}\left(f\right)=X_{s}\left(f\right)\sum_{k=0}^{N-1}\exp\left(-i2\pi fkT_{aq}\right).$$

Clearly, since $X_{s}\left(f\right)$ is band-limited, so it is $X_{aq}\left(f\right)$. The last summation is also a known result, it being the DFT of the discrete-time rectangular function with *N* samples:

(A8) $$\begin{array}{c} \sum_{k=0}^{N-1}\exp\left(-i2\pi fkT_{aq}\right)=\exp\left[-i\pi f\left(N-1\right)T_{aq}\right]\frac{\sin\left(N\pi fT_{aq}\right)}{\sin\left(\pi fT_{aq}\right)}=\\ =\exp\left[-i\pi f\left(T_{s}-T_{aq}\right)\right]\frac{N_{aq}}{N_{s}}\frac{\mathrm{sinc}\left(fT_{s}\right)}{\mathrm{sinc}\left(fT_{aq}\right)} \end{array}.$$

Thus, finally:

(A9) $$\frac{1}{N_{s}}X_{s}\left(f\right)=\frac{1}{N_{aq}}X_{aq}\left(f\right)\exp\left[i\pi f\left(T_{s}-T_{aq}\right)\right]\frac{\mathrm{sinc}\left(fT_{aq}\right)}{\mathrm{sinc}\left(fT_{s}\right)},$$

i.e., the same result is obtained as that in (A4) and (10).
